# Mesenteric ischemia caused by chronic occlusion of multiple mesenteric arteries: a case report

**DOI:** 10.1186/s40792-024-02013-6

**Published:** 2024-09-06

**Authors:** Qin-Ming Zhao, Zhong-You Xu, Hui Wang

**Affiliations:** Department of Vascular Surgery, The Affiliated People’s Hospital of Ningbo University, Ningbo, Zhejiang China

**Keywords:** Mesenteric artery occlusion, Mesenteric artery ischemia, Angioplasty, Stent

## Abstract

**Purpose:**

Chronic mesenteric ischemia (CMI) is a rare disease that progresses with acute mesenteric ischemia, along with high mortality. How to choose the appropriate surgical method and the artery which should be opened first is the key to the treatment.

**Case report:**

In this study, we successively used vascular bypass and endovascular therapy to treat a case of complex chronic mesenteric ischemia.

**Conclusion:**

For mesenteric ischemic disease, the superior mesenteric artery (SMA) should be opened preferentially. Arterial bypass or interventional therapy can be used, or both can be combined, to finally achieve the purpose of treatment.

## Introduction

CMI is a rare disease, with an incidence rate of 9.2/100000 [1]. It is mainly caused by arteriosclerotic occlusion, and other rare causes include arterial dissection, myofibrillar dysplasia, and arteritis major. CMI is often called “intestinal angina.” Typical symptoms are recurrent postprandial abdominal pain and weight loss, leading to food phobia [2, 3]. However, the typical triad is only seen in 16–22% of patients [4]. CMI caused by severe stenosis or chronic occlusion of the SMA is the most common. If stenosis or occlusion of the celiac trunk artery and inferior mesenteric artery (IMA) are combined, the clinical manifestations are more prominent. Therefore, restoring the blood supply of the SMA is the key to treating or relieving symptoms. A case of SMA, celiac artery (CA), and IMA occlusion is reported.

## Case

**Fig. 1:**
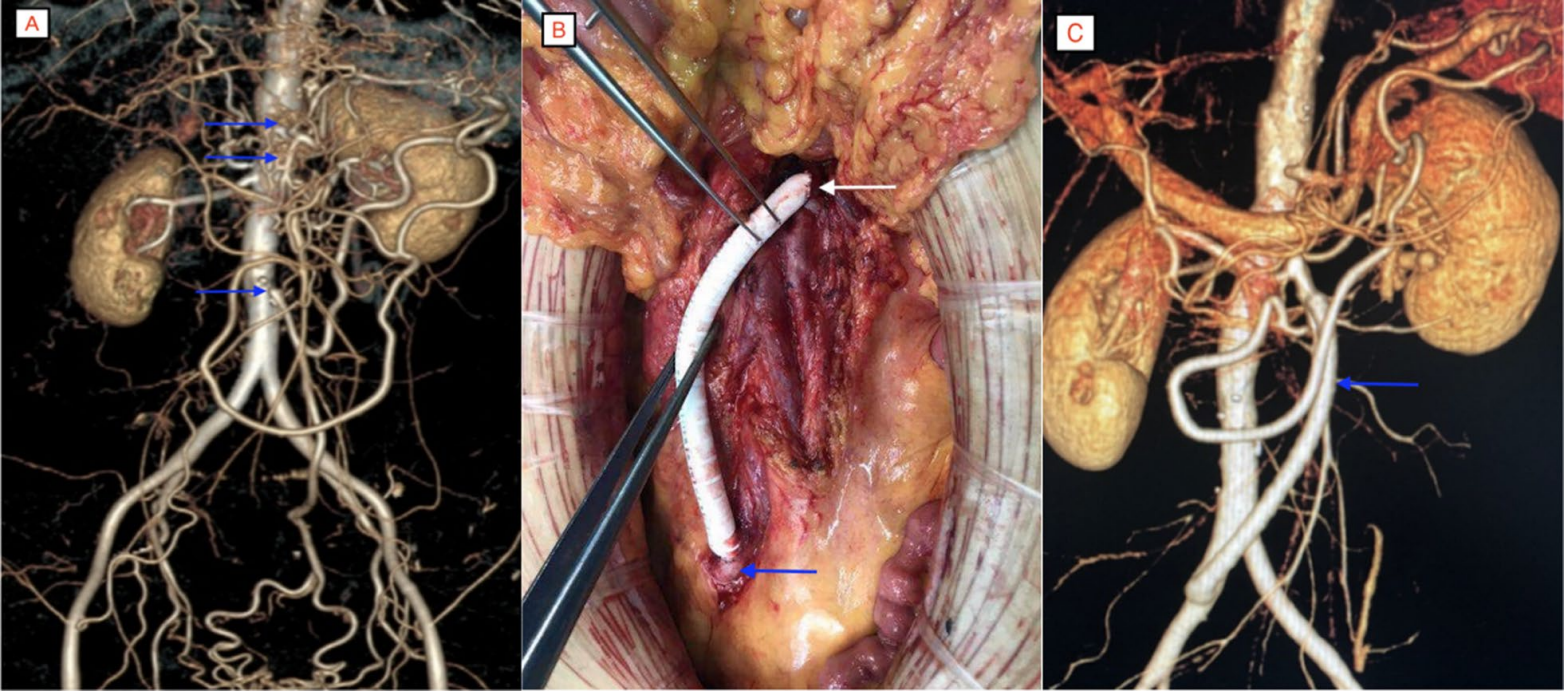
**A** SMA, CA, and IMA were occluded (blue arrow) with many collateral vessels. **B** Artificial blood vessel bypass, SMA anastomosis (white arrow), and right common iliac artery anastomosis (blue arrow). **C** Postoperative CTA image, artificial blood vessel patency (blue arrow)

**Fig. 2:**
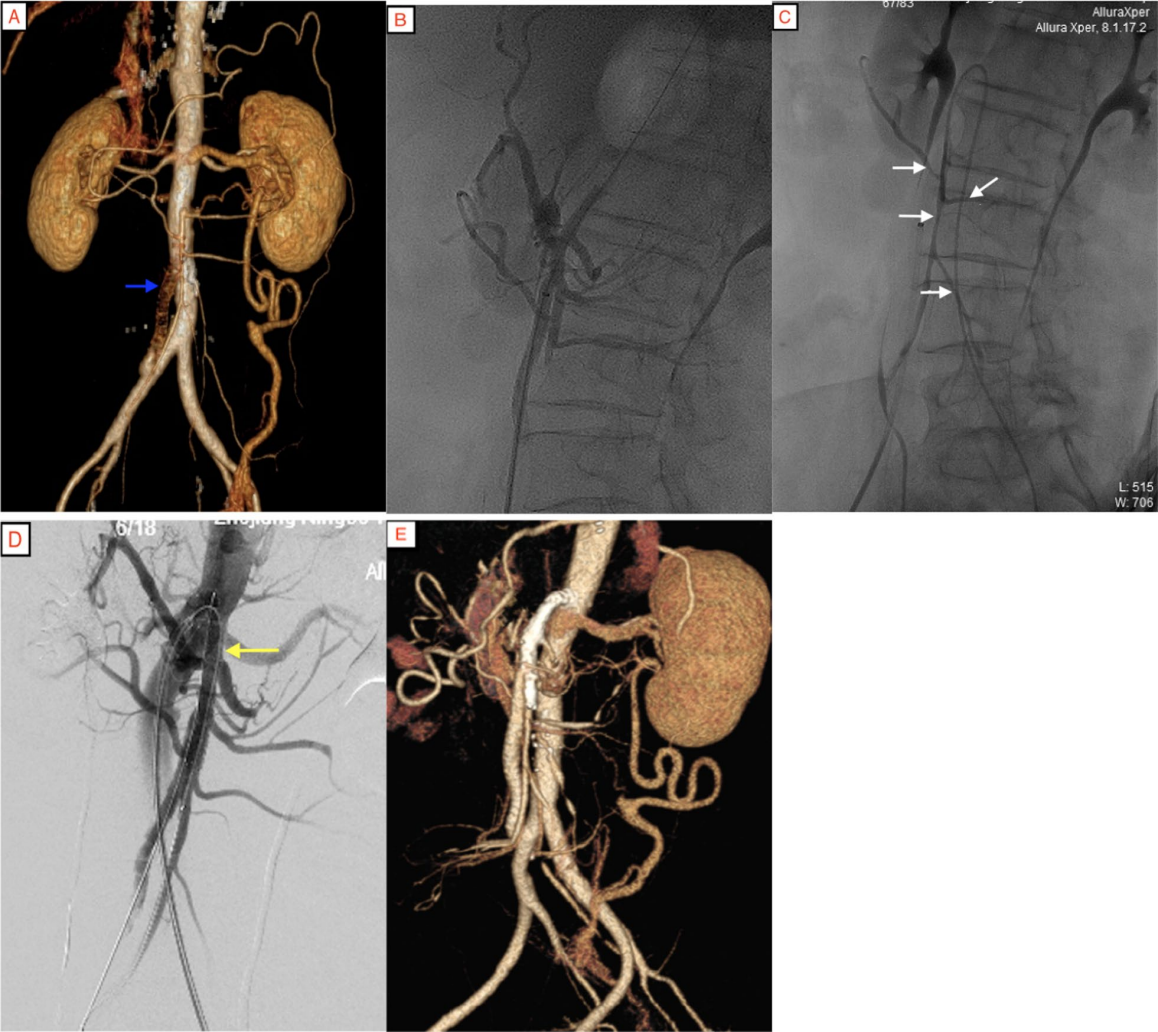
**A** Artificial vessel occlusion (blue arrow), considering thrombosis. **B** The guidewire passes through the occluded SMA segment to the abdominal aorta. **C** SMA and branch development (white arrow) are seen in anterograde angiography through left femoral artery catheterization. **D** The blood flow returns smoothly after SMA stent (yellow arrow) is placed. **E** CTA reexamination after surgery

A 61-year-old woman had pain around the umbilicus for 4 years, which had worsened for half a year. She was referred from the external hospital to our hospital. On admission, she showed the typical triad of chronic SMA occlusion: postprandial pain, anorexia, and weight loss. Her weight had decreased by 20 kg in 4 years. She had no other history of chronic disease or surgery. Computed tomographic angiograph (CTA) examination of the abdominal aorta in the external hospital showed that the openings of the celiac trunk, SMA, and IMA were occluded, accompanied by a large number of collateral arteries (Fig. 1A). After all preoperative preparations, digital subtraction angiography was performed to confirm that the openings of the three mesenteric arteries were completely occluded. Endovascular therapy was abandoned due to no access, and we changed to open surgery. We chose a retrograde bypass because the operation is relatively simple. The operative method involved bypass grafting between the middle segment of the SMA and the right common iliac artery (Fig. 1B). After the operation, the patient’s abdominal pain was relieved quickly, and her weight increased. CTA showed that the artificial vessel was unobstructed (Fig. 1C). After the operation, rivaroxaban tablets 10 mg/day and aspirin tablets 100 mg/day were taken orally. However, half a year later, the patient suddenly experienced abdominal pain, and CTA showed thrombosis in the artificial blood vessel (Fig. 2A). Percutaneous mechanical thrombectomy was performed on the artificial vessel. During the operation, the guidewire reversely passed through the SMA opening to the abdominal aorta through the artificial vessel (Fig. 2B, C). Further balloon dilation and SMA stent placement (1 Lifestent 8 × 60 mm bare stent, Bard, USA; Fig. 2D) were performed. After long-term double-antibody treatment (75 mg/day clopidogrel bisulfate tablets and 100 mg/day aspirin tablets), the patient had no abdominal pain after regular outpatient reexamination (Fig. 2E).

## Discussion

Chronic mesenteric artery ischemia can lead to severe malnutrition, even intestinal ischemia and necrosis, endangering the patient’s life [5]. The natural history of symptomatic CMI patients shows that 20–50% of patients will develop life-threatening acute mesenteric ischemia [6]. Therefore, revascularization surgery is of great value [7]. The intestinal blood supply mainly comes from the CA, SMA, and IMA, and abundant collateral circulation exists among them. The traditional view is that CMI symptoms can only occur if two or more main blood supply vessels have stenosis [8, 9], but there are also views that ischemia can also occur if one vessel, especially the SMA, is narrowed or occluded.

The surgical treatment of CMI includes vascular bypass and endovascular intervention. Arterial bypass surgery may be anterograde or retrograde. The former has the advantages of anterograde bypass surgery and a high patency rate [2], but it is relatively difficult to expose, is difficult to operate, and has a high incidence of complications. The latter has the advantages of a simple operation, but it involves reverse blood flow and a high risk of re-occlusion. Endovascular interventional therapy has the advantage of being minimally invasive. It can be implemented under local anesthesia with low complications and mortality. In recent years, it has gradually become the preferred treatment method for CMI [10]. Therefore, as long as the diseased artery has a “stump” on arteriography, anterograde interventional therapy can be attempted. If the opening is completely occluded and the opening position of the mesenteric vessels cannot be identified, reverse or two-way approaches can also be tried under the intervention [11–16]. If two-way patenting is carried out through the marginal artery or Riolan artery arch, of course, this method requires a precondition. At least one CA has no obvious stenosis, which can be used as an approach for reverse patenting. For units with hybrid operating rooms, it can also be considered to open the normal lumen in the middle and far segments of the SMA and conduct retrograde puncture under direct vision to recanalize the occluded vessels at the ostium. Due to the lack of a hybrid operating room, we were unable to perform retrograde open mesenteric stent implantation surgery initially and opted for bypass surgery.

In this case, for the first time, we used reverse artificial vessel bypass for open surgery, but the thrombosis in the artificial vessel was re-occluded half a year later, indicating that the patency of reverse bypass was not high. Therefore, during the second operation, we tried to recanalize reversely the occluded segment of the opening of the SMA through the artificial vessel, place the stent in parallel, and open positive blood flow. No re-occlusion occurred in the follow-up 2 years. The abdominal pain symptoms never recurred, indicating that the patency of positive blood flow remained good. When two or more of the CA, SMA, and IMA have stenosis or occlusion, opening the SMA preferentially can effectively alleviate the symptoms of intestinal ischemia [17], which was confirmed in this case.

For the selection of stents for lesions at the opening, balloon dilation stents should be preferred, which have the advantages of accurate positioning and strong radial support. In this case, due to the limited equipment varieties, self-expanding bare stents were used, resulting in inaccurate positioning and long stent protrusion into the abdominal aorta.

## Conclusion

Based on this case, the following suggestions are summarized: 1. For multi vessel chronic mesenteric artery occlusion lesions, prioritizing the opening of the SMA can effectively alleviate mesenteric ischemia symptoms; 2. For hospitals with hybrid operating rooms, intracavitary treatment can be attempted first. If unsuccessful, open surgery can be considered to assist in removing the normal lumen of the SMA downstream, and retrograde puncture can be performed under direct vision to open the occluded segment of the artery. Arterial bypass surgery is the last resort, so hybrid operating rooms have advantages in treating complex CMI cases. Figure 1A SMA, CA, and IMA were occluded (blue arrow) with many collateral vessels. B Artificial blood vessel bypass, SMA anastomosis (white arrow), and right common iliac artery anastomosis (blue arrow). C Postoperative CTA image, artificial blood vessel patency (blue arrow) Fig. 1A SMA, CA, and IMA were occluded (blue arrow) with many collateral vessels. B Artificial blood vessel bypass, SMA anastomosis (white arrow), and right common iliac artery anastomosis (blue arrow). C Postoperative CTA image, artificial blood vessel patency (blue arrow) Fig. 1A SMA, CA, and IMA were occluded (blue arrow) with many collateral vessels. B Artificial blood vessel bypass, SMA anastomosis (white arrow), and right common iliac artery anastomosis (blue arrow). C Postoperative CTA image, artificial blood vessel patency (blue arrow)

## Data Availability

The data sets generated and/or analyzed in the current study are available from the corresponding author upon reasonable request.

## Abbreviations

CMI: Chronic mesenteric ischemia
SMA: Superior mesenteric artery
IMA: Inferior mesenteric artery
CA: Celiac artery
CTA: Computed tomographic angiograph
